# Understanding Pride in Eating Disorders and Disordered Eating Behaviors: A PRISMA-Informed Scoping Review

**DOI:** 10.3390/jcm15020522

**Published:** 2026-01-08

**Authors:** Edoardo Del Conte, Lucia Tecuta, Elena Tomba

**Affiliations:** Department of Psychology “Renzo Canestrari”, University of Bologna, Viale Carlo Berti Pichat, 5, 40127 Bologna, Italy; edoardo.delconte2@unibo.it (E.D.C.); lucia.tecuta2@unibo.it (L.T.)

**Keywords:** pride, eating disorders, self-conscious emotions, anorexia, bulimia

## Abstract

**Background**: Pride may play a role in eating disorders and related symptomatology. This PRISMA-informed scoping review explores the emotion of pride in eating disorders and in related symptoms. **Methods**: Four databases (PubMed, PsycINFO, Web of Science, Scopus) were searched (July 2025). This systematic scoping review followed PRISMA guidelines and PICOS procedures. The included reports were written in English language and assessed pride in relation to eating disorder-related symptoms both in diagnosed individuals and in the general population. **Results**: Twenty-three studies were selected, including sixteen quantitative and seven qualitative studies. Pride was evaluated in various forms, including appearance-related pride, body pride, and LGBT pride. In general population samples, high levels of maladaptive pride and low levels of adaptive pride were associated with greater eating disorder-related symptomatology. In patients, pride was associated with control and illness identification and was found to contribute to illness onset, maintenance, and recovery. **Conclusions**: Integrating pride experiences in the assessment of eating disorders may be clinically useful and provide guidance for treatment planning. Main gaps consist in the great heterogeneity of the instruments used to assess pride and in the major focus on anorexia nervosa, with only a few studies investigating bulimia nervosa and binge-eating disorder.

## 1. Introduction

Eating disorders (EDs) are among the most complex psychiatric disorders, carrying high rates of both medical and psychiatric comorbidities. While several evidence-based psychological treatments are recommended in international clinical guidelines, such as the National Institute for Health and Care Excellence [1] and the American Psychiatric Association [2] guidelines, treatment outcomes remain non-optimal, with efficacy rates of around 40–60% [3] in addition to high rates of residual symptomatology persisting even after standard treatment [4]. Moreover, relapse rates remain high with meta-analytic data [5], finding a relapse rate of 31% for anorexia nervosa (AN) and longitudinal studies reporting high relapse rates in bulimia nervosa (BN) as well, with roughly 70% of patients relapsing [6].

To improve treatment outcomes and to deepen the understanding of the clinical course of ED pathologies, a clinical assessment exclusively relying on a conventional ED diagnostic classification systems is limiting. A standard diagnostic assessment does not provide sufficient clinical information, hampering efficacious treatment planning and limiting definitions of outcomes to a restricted set of ED-specific psychopathological criteria [7,8]. Thus, ED researchers have called for extending the definition of outcomes beyond the core symptomatology of EDs, including also non-ED specific psychological features which appear to have great salience in the psychopathological network models of these disorders [9].

One area warranting inclusion as core in ED-related symptomatology is represented by affectivity [9]. Negative emotions and emotion dysregulation have been found to play a key role in the onset and maintenance of EDs, despite not being typically considered core ED-related symptomatology [10,11,12,13]; however, positive emotions are rarely considered [14]. Recently, self-conscious emotions (SCEs) have captured particular attention in ED research and appearance-related aspects such as body and weight [15,16,17,18].

SCEs are those emotions that are intimately connected with the sense of self and thus with the appraisal process involving cognitive mechanisms of self-evaluation and self-reflection [19,20]. If the appraisal of the self, including attributes and behaviors, is negative, then this may result in unpleasant emotions including shame and guilt while, if positive appraisals are made, pride may be experienced [17].

Among SCEs, pride [20,21,22] in particular might play an important role in EDs. Pride experienced in the process of identification with the illness, especially in patients with AN [23], or a lack of pride in other EDs—mainly BN and binge-eating disorder (BED)—could represent a maintenance factor, a facilitator, or a severe obstacle to treatment engagement. Rebuilding a healthy sense of identity, detached from the illness, has indeed recently been identified by several qualitative studies as a key factor in the recovery process from EDs [24,25]; for example, in a qualitative systematic review, Wetzler et al. found that recovering a healthy sense of identity was a turning point in the recovery process and one of the most difficult things to obtain [26]. According to the current literature, SCEs, being so intimately intertwined with the sense of self, can be important in the construction of one’s identity [17,22]. It is noteworthy that pride can be broken down into two distinct components: authentic and hubristic [27]. Authentic pride is linked to feelings of accomplishment (such as success and ability), improved self-esteem, positive personality traits, and motivation towards goal-oriented behavior [27,28,29,30,31]. On the other hand, hubristic pride is associated with narcissistic behavior, such as arrogance and conceit. Both types of pride have both adaptive and maladaptive functions [27,28,29,32]. While other emotional experiences, such as shame and guilt, have been extensively researched in recent years, pride has received far less attention [33,34,35,36]. Despite its potential relevance in ED onset, maintenance, and recovery, pride remains relatively unexplored and, to date, no systematic review has been conducted on this emotion. Deepening our knowledge on the role of pride in ED patients and in relation to ED-related symptoms could aid both researchers and clinicians in improving treatment planning and outcomes.

The current study consists in a scoping systematic review which investigates the extent of the literature exploring the presence and the clinical characteristics of pride in ED patients and its possible associations with ED psychopathology. We established the following research questions:

- What are the characteristics of studies investigating pride in EDs?
- What are the most commonly used instruments or measurement procedures to assess pride?
- What has the literature found about the relation between pride and EDs and between pride and ED-related symptomatology?
- Is there evidence supporting the role of pride in the onset, maintenance, and recovery of EDs?

In particular, this scoping review will provide an overview of the existing literature concerning the relation between pride and ED-related symptomatology in both ED patients and in general population samples assessed for ED-related symptoms and behaviors.

## 2. Methods

The present review was conducted following the Joanna Briggs Institute scoping review protocol [37], the Preferred Reporting Items for Systematic reviews and Meta-Analyses (PRISMA) criteria guidelines [38], and the PRISMA extension for scoping systematic reviews [39]. A scoping review was deemed appropriate as currently available studies are few in number and heterogeneous in nature, making a conventional systematic review not possible at the moment. Both the extended PRISMA checklist and the Scoping Review checklist can be found in the Supplementary Materials. The review protocol was registered at the Open Science Framework [40] (registration number: d53mx).

Four databases (Scopus, PubMed, PsycINFO, Web of Science) were systematically searched (up until July 2025) for original, peer-reviewed articles using the keywords “anorexia”, “bulimia”, “binge-eating”, “binge eating”, “eating disorders”, and “disordered eating”, each separated by the Boolean operator OR and matched to the keyword “pride” via the Boolean operator AND. No time limits were added to explore the evolution of the research on the topic over time. The results were filtered through the English language; no temporal limits were applied. Titles and abstracts were screened by one author (E.D.C.). Articles that appeared to be potentially relevant were retrieved and reviewed by three researchers (E.T., L.T., and A.P.) who independently assessed each of the full reports. When disagreements between the three authors arose, multiple rounds of full-text revision and discussions were undertaken until a consensus was reached.

### 2.1. Eligibility Criteria

The eligibility criteria for this review are reported using the Population, Intervention, Comparison, Outcome, Study (PICOS) type model [41,42] and are shown in Table 1. Eligible articles were written in the English language. The following types of articles were included: observational and quasi-experimental studies, longitudinal studies, cross-sectional studies, clinical trials, and case reports. Grey literature, opinion studies, book chapters, and unpublished studies were excluded. Any study which assessed or explored the construct of pride through psychometric questionnaires or qualitative interviews in ED patients or concerning ED-related symptoms in general population samples was included in the review. Studies were excluded if the sample was not composed of ED patients or if no questionnaire was employed for assessing ED-related symptoms.

### 2.2. Data Extraction and Synthesis

Data were extracted from papers included in the scoping review using a data extraction table developed by the authors (see Table 2). The data extracted included specific details about the participants, as well as study characteristics and key findings relevant to the review questions. Relevant findings extracted for each study on pride in ED patients and about ED-related symptoms includes the following: mean age in years, percentage of female participants, sample size and type (ED patients or general population sample) with ED diagnosis when available, study type (qualitative or quantitative), instruments used to measure pride and to assess EDs or ED-related symptoms, study design, main outcomes about pride, EDs, and ED-related symptomatology (see Table 2). Any disagreements about data extraction that arose between the authors were resolved through multiple rounds of full-text revision and discussions until consensus was reached. If needed, the authors of included reports were contacted to request missing or additional data. The review included both quantitative and qualitative studies, therefore the synthesis was conducted using an integrated synthesis protocol [43].

## 3. Results

**Figure 1:**
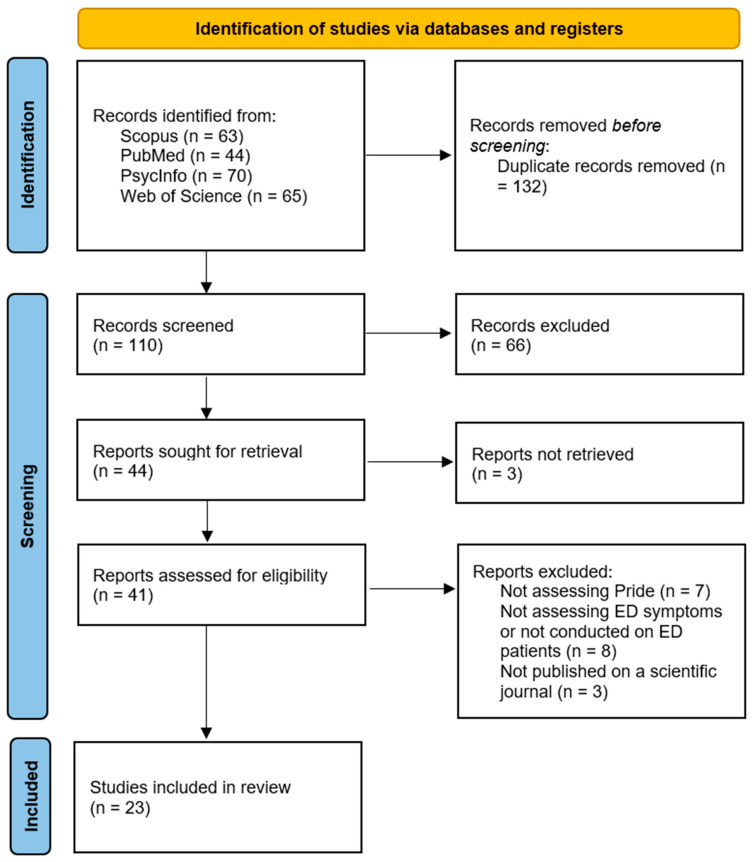
PRISMA flow diagram for the research process.

The research produced 63 results in Scopus, 44 in PubMed, 70 in PsycINFO, 65 in Web of Science, and 1 result through a manual search, for a total of 242 records of which 132 were duplicates. After title and abstract screening of the remaining 110 results, 66 were dismissed. Of the remaining 44 articles, 3 reports were not retrieved and an additional 18 reports were dismissed as they did not meet the inclusion criteria (specifically: 7 records did not assess the construct of pride, 8 records were not conducted on ED patients nor investigated ED-related symptoms or behaviors, and 3 records were not published in peer-reviewed scientific journals), for a total sum of 23 articles included in the review. The study selection process is summarized in the PRISMA [38] flow diagram (see Figure 1). A reference search was conducted manually to find other eligible articles, but no other records were included.

### 3.1. Review Question 1: Study Characteristics

Of the 23 included studies, 12 studies (52.2%) investigated pride in various types of general population samples, 2 studies (8.7%) focused on LGBTQIA+ populations, 1 study (4.3%) focused on patients with chronic illnesses, while the remaining 8 studies (34.8%) focused on ED patient samples (7 studies on AN, 1 study on mixed EDs). In terms of study design, 7 studies (30.4%) are qualitative, while the other 16 studies (69.6%) are quantitative. To summarize the quantitative results, despite the heterogeneity in the instruments and statistical methods used to assess the role of pride and the extent of eating-related symptomatology, a summary table has been developed (see Table 3).

The seven qualitative studies employed samples ranging from 5 to 21 participants and focused on clinical ED samples, the majority (six studies) considered AN patients [45,47,49,51,60,62] and one considered a mixed ED sample with self-diagnoses of AN, BN, and BED [57]. Two out of the seven qualitative studies [57,60] did not report gender percentages, while the other five studies [45,47,49,51,62] employed a mostly female sample (from 87% to 100% females). Age range in the qualitative studies varied widely, ranging from 16 to 50; not all studies reported mean age due to limited sample sizes. The qualitative studies were either grounded theory or descriptive phenomenology-based studies. Several studies used open-ended [47], in-depth [60], semi-structured interviews [45,49,51,57,62] with thematic analyses or other methods of text analyses.

The 16 quantitative studies employed larger samples (ranging from 23 to 17,159 participants) compared to the qualitative studies. Of the 16 studies, 1 study recruited individuals with an ED (AN) diagnosis [53], 2 studies recruited LBTQIA+ individuals, respectively transgender/gender expansive (TGE) people or LGBTQ adolescents [52,58], while the remaining 13 studies recruited various general population samples, including participants with chronic illnesses and ED-related symptoms, or recruited from pro-anorexia communities and recovery websites. Eight studies had a fully female sample [46,48,50,53,55,63,64,66], while four studies [44,54,59,61] had a more balanced sample, with the percentage of female participants ranging from 41% to 77.3%. One study [56] was conducted on men; for the two studies conducted on LGBTQIA+ population samples [52,58], the female percentage was not applicable. Finally, one study [65] did not report gender percentages.

The age range varied among the quantitative studies as well (from full text readings, age ranges from adolescence to over 70 years), although two studies [50,60] did not report exact age measures. The majority of the quantitative studies were cross-sectional, while one study employed a longitudinal non-randomized clinical trial design [65] and three studies employed ecological momentary assessment (EMA) to investigate pride [53,63,66]. Additional characteristics of the selected studies can be found in Table 2. No controlled studies with comparisons on pride between ED patients and general population samples or non-ED controls emerged.

### 3.2. Review Question 2: Most Commonly Used Instruments or Measurement Procedures for Pride

Concerning the seven qualitative studies [45,47,49,51,57,60,62], the measures used to investigate pride were interviews (unstructured or semi-structured), but no study reported the specific questions/items.

Concerning the 16 quantitative reports, the only quantitative study on an ED (AN patients) population [53], employed the State Shame and Guilt Scale (SSGS [67]), a self-report instrument developed for the measurement of shame and guilt, which however includes a subscale (five items) for the measurement of pride (e.g., “I feel good about myself” and “I feel capable, useful”). Among the remaining 15 quantitative studies that involved non-ED populations, 2 studies [61,65] also employed the SSGS [67], respectively in patients with chronic illnesses and in the general population.

One study consisted in the validation of a novel ED-specific measure for pride, called the Pride in Eating Pathology Scale (PEP-S [48]), which identifies various facets of ED-specific pride; the instrument is composed of a total score and four subscales, namely “Pride in weight loss, food control and thinness”, “Pride in healthy weight and healthy eating”, “Pride in outperforming others and social recognition”, and “Pride in capturing other people’s attention due to extreme thinness”.

Seven studies focused on appearance-related pride aspects and measures. In particular, one study [50] employed and validated the Body Pride and Shame scale (BPS), which specifically measures body pride, both current and anticipated; one other study [63] used the Body-related Self-conscious Emotions Fitness instrument (BSE-FIT [56]), an instrument specifically developed to assess fitness-related SCEs. The BSE-FIT includes two scales focusing on hubristic and authentic body pride where authentic pride is based on specific, controllable achievements and behaviors (e.g., “I finished the marathon I trained for”), whereas hubristic pride is linked to global aspects of the self (e.g., “I am a fit person”) typically involving feelings of personal grandiosity and superiority to others [28,30].

Four studies [54,55,56,63] employed the Body and Appearance Self-conscious Emotions Scale (BASES [15]), although one of the studies [63] employed just one item for each subscale to be able to administer it into an EMA design. The BASES is a four-factor measure that investigates body- and appearance-related shame, guilt, and both authentic and hubristic pride, each with four items on a five-point Likert scale. One study [59] employed two different scales to assess authentic pride: the Body Related Pride Scale (BRPS [30]) and the authentic subscale of the Authentic and Hubristic Pride Scale (AHPS [27]).

One study [58] used a subscale of the Gender Minority Stress and Resilience Measure (GMSR [68]) that specifically investigates gender-related pride, conceptualized as a gender-related resilience factor. Two studies [46,50] employed ad hoc non-validated instruments; in particular, Cornelius and Blanton [46] recruited participants from pro-anorexia and ED recovery websites and used an ad hoc Likert scale questionnaire to assess pro-anorexia pride, while French et al. [50] employed an ad hoc non-validated structured interview with a Likert scale, seemingly clinician-administered.

One study [66] employed Visual Analog Scales (VAS) in an EMA design to investigate feelings of pride related to fasting: participants were asked to complete the measures at a baseline and then every 2 h throughout fasting and non-fasting days, with self-report items ranging from 1 to 10 (ported items are, for example, “Sense of pride” and “Sense of achievement”). Finally, one study [52] employed ad hoc self-report questionnaires to investigate specifically LGBTQ pride, with questions such as “I am proud to be part of the LGBTQ community”.

The instruments employed in each study can also be found in Table 2.

### 3.3. Review Question 3: Pride in Relation to EDs and to ED-Related Symptoms

#### 3.3.1. Pride in Non-ED Samples

While qualitative studies only investigated ED samples, 15 of the 16 quantitative studies were conducted on non-ED samples (65% of the total included studies). Of these fifteen studies, one was conducted on pro-anorexia communities and ED recovery website members [46], two on LGBTQIA+ samples [52,58], one on MTurk (an Amazon crowdsourcing platform) workers [59], and one on patients with chronic illnesses [65], while the other ten were conducted on general population samples.

Of these fifteen quantitative studies on non-ED populations, eight (53%) found positive associations between some facets of pride (authentic and hubristic pride, pro-anorexia pride, ED-related pride, and anticipated body-pride in weight loss) and ED-related symptoms; inversely, seven studies (47%) found negative associations between other forms of pride (LGBTQ pride and authentic and hubristic body-pride) and ED-related symptoms. Summarized results can be found in Table 2 and Table 3.

Specifically, concerning the positive associations between pride and ED-related symptoms (greater levels of pride associated with greater levels of disordered eating), Alcaraz-Ibáñez et al. [44] found that both hubristic and authentic pride explained morbid exercise behavior (MEB), while adjusting for age, sex, and disordered eating symptoms, and authentic pride also explained exercise frequency. Moreover, the direct relationship between pride and MEB was weaker (when hubristic pride was considered) or stronger (when authentic pride was considered) in participants with higher levels of disordered eating symptoms. In the study by Cornelius and Blanton [46], a positive correlation between maladaptive, pro-anorexia pride and disordered eating behaviors was reported in individuals recruited from pro-anorexia and ED recovery websites, where the endorsement of pro-anorexic beliefs also predicted lower self-esteem. In this study, the endorsement of pro-anorectic beliefs and the identification of patients with values associated with AN (e.g., perfectionism, perseverance, etc.) made patients feel proud, albeit lowering self-esteem, and reinforced their identification with the disorder.

Faija et al. [48] reported significant and positive correlations between ED-related pride and ED psychopathology in terms of eating restraint and eating, weight, and shape concerns. Pride in ED aspects characterized participants from the general population with high ED-related symptomatology. Moreover, the global score for ED-related pride and the subscales of “pride in weight loss”, “pride in social recognition”, and “pride in capturing attention” of the PEP-S were negatively correlated with authentic pride and self-esteem; however, the subscale “pride in healthy eating” was not associated with ED-related symptoms and was positively associated with both self-esteem and authentic pride. Marques et al. [54] adopted a network approach to investigate the interaction of pride with ED-related symptoms, and found a high centrality of the pride nodes, both authentic and hubristic appearance-related pride. More specifically, the results highlight a relationship between authentic pride and cognitive restraint, suggesting that successful restraint may trigger feelings of authentic pride and a relationship between hubristic pride and social comparison through appearance. The authors point out that this relationship between pride and ED-related symptoms generate a vicious cycle, reinforcing the identification with the illness and contributing to the maintenance of the disorder.

Muratore et al. [58] investigated a specific form of pride in gender minority pride, conceptualized as a resilience factor; contrary to the researchers’ hypothesis, pride showed positive correlations with both thinness- and muscularity-oriented disordered eating. The employed measure, the GMSRS, focuses especially on transgenderism, with items such as “It is a gift that my gender identity is different from my sex assigned at birth” and “I am proud to be a person whose gender identity is different from my sex assigned at birth”. The subscale also showed correlations with minority stressors, like internalized transphobia, which are in turn associated with disordered eating, suggesting a possible mediated effect which was not investigated in the paper.

Sandoval-Araujo et al. [61] investigated the possible moderator role of pride in the relation between ED-related symptoms and social appearance anxiety (SAA), finding that, with lower levels of pride with no distinction between authentic or hubristic, the correlation between SAA and ED-related symptoms was stronger. Troop [64] investigated current and anticipated body pride (i.e., pride anticipated to experience if the participants were to gain or lose weight), finding associations with dietary restraint and low caloric intake, where having higher levels of pride in anticipating a weight loss predicted restriction in terms of lower caloric intake over the next week. The last of the quantitative studies that found detrimental effects of pride [66] employed an EMA design, investigating mood and affect fluctuations in healthy women when inducing (and when abstaining from) fasting. Consistent with the hypotheses, researchers found that participants reported a higher sense of pride, achievement, and control at the end of the fasting period compared to both the start of the fasting period and the end of the non-fasting period.

Concerning the negative associations between pride and ED-related symptoms (i.e., to higher pride corresponded lower disordered eating), French et al. [50] found that high levels of body pride coincide with lower levels of disordered eating behaviors. Lawrence et al. [52] investigated the role of LGBTQ pride, similarly to the previously cited studies from Muratore et al. [58], finding opposed results: adolescents with bias-based bullying and with lower pride were more likely to engage in disordered eating behaviors, although the biggest difference seemed to be made by bias-based bullying. Three studies [55,56,63] employing or adapting the BASES [15] found that hubristic and authentic body pride had a protective role in relation to ED-related symptoms, either directly [56] or indirectly through other related constructs, namely appearance comparison [63], emotion regulation [56], and symptoms of depression and anxiety [55]. Moreover, individuals considered at risk for an ED were found to have significantly lower hubristic and authentic body pride [56].

Nesbitt et al. [59] investigated only authentic facets of pride, both global and body-related, and found a positive association between global authentic pride and mental health indices, such as self-esteem and flourishing, and a negative association between body-related authentic pride and eating pathology. Lastly, one study [65] found that pride was significantly improved, while shame, guilt, and ED-related symptomatology were decreased after a short psychoeducational CBT-based group intervention meant to improve eating concerns and pathology in chronic medical conditions (including diabetes type II, hypertension, and high cholesterol), pointing towards a negative correlation between pride and eating symptomatology.

#### 3.3.2. Pride in ED Patients

Among the eight studies on ED patient samples, seven (87.5%) involved patients with AN, while one (12.5%) focused on a mixed-ED sample. While one study was quantitative, the remaining six studies on ED samples were qualitative.

The only quantitative study [53] found positive associations between pride and ED-related symptomatology, finding in AN patients a positive relation between SCEs, perceived social rank, and exercising. Their findings support how weight-control behaviors (dieting, purging, hyperactivity) momentarily raise perceived social rank, usually low in ED patients, due to increased feelings of pride and decreased feelings of shame immediately after exercising.

Across the remaining six qualitative studies, several common themes about associations between pride and EDs emerged. A recurrent result concerns pride and anorexic status, specifically the patients’ perception of AN as the most desirable of all the EDs, described by patients as a “much bigger, better and truer” experience compared to being “normal” [62]. Included studies found a proper hierarchy of EDs, with AN being considered among patients as “the top one … [because] you’re an exemplar of resilience and determination and you work hard” [57]. This perceived superiority of AN seems linked positively both to personality traits (e.g., perfectionism) and body shape [49,57,60]. Another result common to all the included qualitative studies [47,49,51,57,60,62] concerns control and pride. On the one hand, a perceived loss of control in life, which seems to precede the onset of AN and to persist throughout the disorder, has been highlighted as intertwined with pride when patients prove themselves capable of controlling food, weight, and body shape: “Their self-starvation assuaged the sense of chaos” [47]. On the other hand, a loss of control over food, weight, or body shape, or the inability to meet unreasonably high standards, is associated with shame. Pride may serve as a means to suppress feelings of shame, and the two emotions combined have a role in the maintenance of EDs, especially in AN [51,57,62]. In the case of severe and enduring AN (SEED-AN), pride is achieved not only by controlling food but by controlling one’s own disorder: the capacity of living with AN for so many years (more than 20 in the study) is a source of perceived resilience and positive emotions [60].

### 3.4. Review Question 4: Pride in the Onset, Maintenance, and Recovery of EDs

In five qualitative studies on ED patients, the role of pride at the onset emerged mostly in AN patients [47,48,51,60,62]. In particular, the qualitative data suggest that, when the person begins to lose weight, compliments and attention by peers or by significant others elicit pride, characterized by a pleasant feeling spontaneously experienced and linked to perfectionism and self-imposed standards, social acceptance, and self-control. Moreover, successfully controlling one’s own food intake, body shape, and “enduring” the demands of the illness generates feelings of pride in the early phase of the disorder. This kind of pride has been named “alluring pride” by Faija et al. [49]. This finding is partially supported by quantitative data from the study by Watkins and Serpell [66], who described the increase in feelings of pride during and after fasting periods in healthy women.

Subsequently, when the AN illness becomes increasingly present and demanding, pride experiences become more “toxic”, something to pursue as a way to numb feelings of shame and to reinforce one’s sense of self, intertwined with the concepts of determination and willpower [47,49,51,57,60,62]. This phenomenon was also described in a quantitative study [53], as patients actively pursued pride by adopting weight control behaviors; in particular, the anticipation and immediate moments after exercise were associated with higher levels of pride. Faija et al. [49] finally provided the following definitions: “pathological pride”, when the patient starts to identify with the disorder, and “anorexia pride”, when the level of differentiation from the illness lowers. Data from both the qualitative and quantitative studies are consistent with their conceptualization [46,48,54,57,62].

In their qualitative study, Faija et al. [49] identified three additional forms of pride as more experienced in the recovery process from AN: shameful pride, recovery pride, and resilient pride. “Shameful pride” is experienced when the individual feels ashamed of her/his own pride feelings in pathological achievements and marks the beginning of the progressive differentiation from the illness that occurs during the treatment; “recovery pride” instead constitutes pride in being able to fight one’s own symptoms; while “resilient pride” is a more complex and all-embracing experience which involves changes in the sense of self (a full differentiation from AN) consisting in a new attitude towards one’s self. Both recovery and resilient pride was found by Chinello et al. [45] when investigating AN and pregnancy: the pride experiences that come with becoming a mother played a significant role in the recovery process.

The only qualitative study on mixed EDs described a loss of pride and a loss of positive feelings associated with the disorder emerging during the crossover from one ED diagnosis to another [57]; in particular, in the crossover from AN to BN or BED, the loss of control over food and over body weight and shape is perceived as a shameful experience because it represents a loss of identity and control.

## 4. Discussion

In ED samples, most studies used a qualitative approach and focused primarily on patients with AN. There is limited data on other ED diagnoses, except for mixed samples where diagnostic differences were not analyzed. By contrast, studies on general population samples were quantitative and mostly employed cross-sectional correlational designs. Notably, no studies have provided controlled comparisons of pride between individuals with ED and the general population. This lack of data on between-group differences makes it difficult to determine whether pride, whether dysfunctional and pathological or adaptive and functional, differs between clinical and non-clinical populations. Further research is needed to explore whether different forms of pride are associated with ED psychopathology and how pride may uniquely manifest in individuals with EDs.

Several critical issues concerning assessment emerged in the current review. First and foremost, a vast heterogeneity characterized the instruments used in the available reviewed literature as well as the components of pride that were investigated, revealing a lack of unified and comprehensive operationalization of the construct of pride in EDs which considers its multifaceted nature. On the one hand, a wide range of instruments might offer a more comprehensive and multi-faceted view of pride in EDs and in ED-related symptomatology; on the other hand, it hinders comparability across studies. Specifically, from our data, pride was assessed as a global self-conscious emotion only in the SSGS [67], used in two [53,65] of the selected studies, and in the AHPS [27], used in one of the selected studies [59]. More attention has been paid in the reviewed quantitative literature to appearance-related pride aspects, using the BPS [64], the BSE-FIT [69], and the BASES [15], which are focused on a very specific facet of body pride. In particular, the BPS measures pride related to the actual body and to anticipated body changes (e.g., would you feel proud if you lost/gained weight), the BSE-FIT measures pride in relation to the fitness condition or physical efforts of the participants (e.g., I’m proud of my superior fitness), and the BASES measures authentic and hubristic pride related to perceived appearance and attractiveness (e.g., I’m proud of my appearance accomplishments). Currently, only one standardized instrument has been proposed to measure pride specifically in the context of an eating pathology; that is, the PEP-S [48]. However, so far and during this work, no reviewed study, apart from the validation study itself, has employed it. Further, a specific critical aspect arose in terms of assessment in the seven reviewed qualitative studies [45,47,49,51,57,60,62]. In particular, in relation to the assessment of pride, they did not report the specific interview questions they used to assess pride in their qualitative works, as each study adopted an unstandardized ad hoc interview. A clear understanding of which, if any, specific facets of pride were considered is therefore hampered. Thus, considerations of how pride was conceptualized in EDs across such qualitative studies is not possible.

Despite the heterogeneity in instruments and definitions of pride across studies, a few considerations may be drawn in terms of associations between pride and EDs and ED-related symptomatology. Most of the research supports the association between increased dysfunctional forms of pride and decreased healthy forms of pride with EDs and ED-related symptomatology in both AN patients [45,47,49,51,53,60,62] and in ED patient groups [57], as well as in the general population [44,48,54,58,61,64,66]. Additionally, in a study of individuals recruited from pro-anorexia and ED recovery websites, self-esteem was associated with greater pro-anorexia-related pride [46]. Conversely, in the general population, healthy forms of pride were associated with lower levels of ED-related symptomatology or with protective factors, such as self-esteem [50,52,55,56,59,63,65].

The associations between ED-related symptomatology and increased pathological forms of pride and reduced functional forms of pride can be better explained considering the role of dysfunctional positive emotions in the onset and maintenance of EDs, an aspect which is largely overlooked in the literature, as well as considerations of the intertwined relationship between EDs and identity development [14]. A sense of detrimental self-worth, self-competence, personal mastery, and a sense of achievement may be positively related to the ability of controlling weight in patients with AN [70,71], in line with the long-held clinical notion that themes of mastery and life purpose may be intrinsically linked with the onset of ED-related symptomatology [11,72]. A sense of self-worth and accomplishment might be attributed by such patients to their restrictive behaviors which are interpreted as an ability to obtain control, contributing to a false sense of mastery over one’s self and environment and to a distorted sense of purpose in life [73]. Similarly, the meaning-making model of EDs [74,75] proposes that ED-related symptomatology represents a dysfunctional attempt at assigning, albeit temporarily, through meaning-making processes, a sense of situational meaning to a stressful event that triggered negative emotional states. Moreover, researchers have highlighted the role of pride in identity development where patients tend to ascribe positive attributions to their disorder in defining the meaning of identity [76]. It is interesting to note that, in the current review, one study did not find support for the anorexia–pride hypothesis, according to which disordered eating contributes to a greater sense of self-worth and self-esteem through identification with anorexia, as the endorsement of pro-anorexic beliefs was associated with reduced self-esteem [46].

Finally, in ED patient samples, there is some preliminary evidence, albeit mostly in qualitative studies and focusing on AN, that pride may emerge in association with either onset [47,49,62] or maintenance [47,49,53] and as an obstacle or a facilitator to recovery [49]. In the conceptualization of Faija et al. [49], pride per se does not have a negative or positive influence on EDs, but can serve as a pathological factor (alluring pride, toxic pride) or as a recovery facilitator (recovery pride, resilient pride). In such a complex clinical scenario, a loss of pride associated with a loss of positive emotions has been described as emerging during the crossover from one ED diagnosis to another in a qualitative study on a mixed-ED sample [57], in particular in the crossover from AN to BN or to BED. Nevertheless, most of these studies were cross-sectional and qualitative, thus, more methodologically robust longitudinal studies examining pride in EDs are needed.

## 5. Conclusions

Review question 1: Study characteristics

It is evident that the current research lacks quantitative, longitudinal data on the role of pride in eating disorders, especially in BN and BED. Most quantitative studies have focused on the general population, and none of the included studies have adopted a longitudinal design, except for some niche populations (e.g., the intervention study on patients with chronic illnesses [65]).

Review question 2: Most commonly used instruments or measurement procedures for pride

A great heterogeneity of instruments for the assessment of pride emerged. This result is closely linked to the result of review question 1, as most of the instruments used to assess pride were non-ED-specific, often qualitative, and sometimes not even pride-specific. However, some reliable, specific instruments for the assessment of pride are emerging: in particular, the PEP-S [48] seems to be the most reliable instrument for the assessment of pride in EDs.

Review question 3: Pride in relation to EDs and to ED-related symptoms

All the studies included in the review support some kind of association between pride and ED-related symptoms, sometimes as a protective factor and sometimes as a risk for developing or worsening a condition. In particular, most of the studies agree in the identification of body pride as a protective factor towards the development of ED-related symptoms; however, ED-related symptoms can become a source of pride, possibly initiating the cycle of identification with the illness.

Review question 4: Pride in the onset, maintenance, and recovery of EDs

This brings us directly to review question 4: the studies investigating ED patients agree, both quantitatively and qualitatively, that pride has a role in the onset, the maintenance, the remission, and even in diagnostic crossover. However, the results are still limited by the heterogeneity of the instruments used, the lack of quantitative and longitudinal data, and more generally by the limited number of studies investigating the topic.

### 5.1. Clinical Implications

This scoping review supports the possible relevance of pride, in particular with regards to AN, suggesting that AN treatment might benefit from considering the contribution of pride to the illness and its associations with symptomatology. Interestingly, recent research on EDs and SCEs has been conducted [33], suggesting inversely that overeating-related EDs are linked to shame and guilt more than they are to pride.

Integrating the evaluation of pride in patients with AN may therefore be clinically relevant. For example, as suggested by the authors of [14,77,78], it may be clinically useful to help patients understand how the characteristics and subjective experiences generally considered to be “positive” may be supporting their identification with the illness through meanings they ascribe to their disorder. Moreover, considering low motivation to change in AN, primarily due to the egosyntonic nature and identification with the illness, one strategy that clinicians might adopt is to help patients differentiate themselves from their illness by promoting authentic positive affect, and by undermining dysfunctional emotions as a means to disrupt the vicious cycle of symptom reinforcement. Considering pride as a possible component of their readiness to change and aiding patients to recognize that the disorder had originally been adaptive for the patient might be clinically helpful [79]. However, so far ED-specific treatments [80] have failed to focus on such detrimental aspects of positive affect in EDs, and studies addressing detrimental positive emotions in EDs are needed [81].

### 5.2. Limitations and Future Directions

This review presents several limitations tied to the limitations of the selected studies. The absence of clear geographical details and cultural context in most of the studies represents a minor limitation. The use of the synthesis method that allows a mixed methods review following the Joanna Briggs Institute guidelines [43] does not allow for the same conclusions as a more systematic approach. Given the scarcity of data, greater homogeneity in quantitative studies would be needed. Once quantitative and longitudinal investigations of the role of pride in EDs reach an adequate number, a more systematic or meta-analytic approach could be attempted to specifically address the magnitude of the direct correlation between pride and eating symptomatology in diagnosed patients or the predictive role of pride on ED-related symptoms. Scoping reviews are inherently limited, compared to systematic reviews, in their ability to summarize data, draw conclusions, highlight main gaps and strengths of the current literature; however, they can offer valuable insights into the kinds of research and the potential relevance of understudied topics, such as the one considered in the present study [82]. While the inclusion of studies with qualitative designs provided extensive information about the role of pride in EDs and offered great cues on how to comprehensively operationalize and investigate pride in EDs, they are intrinsically less replicable and more difficult to synthesize. Notably, some of the included studies [47,50,62,65] are 15 or more years old; older studies, especially in qualitative research, can be less reliable, and this can be considered a limitation of both our review and the current literature.

Several major lacunae on pride in the ED literature and in relation to eating symptomatology emerged. Future research should include a specific and reliable instrument for the assessment of pride, such as the recently developed PEP-S [48], as well as on shame, given the close associations with pride, including samples of BED and BN patients. Moreover, controlled studies, which were lacking in the present literature, are warranted to better differentiate ED psychopathology from the general population in pride experiences in its various pathological and healthy facets. Additionally, examining pride in longitudinal studies as well as its associations with ED-related symptomatology and response to treatment would elucidate its potential role in ED maintenance and potential effects on treatment outcomes.

## Figures and Tables

**Table 1:** PICOS model for inclusion and exclusion criteria.

*PICOS*	Inclusion Criteria	Exclusion Criteria	Data Extraction
*Population*	All ages Female, male, or mixed gender studies Patients diagnosed with an ED or general population assessed for ED-related symptoms	Other psychopathologies	Age Percentage female Sample type (ED, general population) ED diagnosis, when present ED symptoms
*Intervention*	Any type of intervention (including absence of intervention)	None	Intervention characteristics when available
*Comparison*	Any type of control group (including absence of control group)	None	Group information when available
*Outcome*	Any aspect of pride, disordered eating, and/or other ED-related symptoms	None	Instruments used to assess pride Instruments used to assess EDs or ED-related symptoms Relationship between pride and development of the illness, when available
*Study design*	Observational and quasi-experimental studies Longitudinal studies Cross-sectional studies Clinical trials Case Reports	Dissertation/thesis Review studies/meta-analyses Book chapters Opinion studies Grey literature Unpublished studies	Type of study (qualitative or quantitative) Design of the study (case study, case-control, cross-sectional longitudinal)

**Table 2:** Characteristics of selected studies.

*Author*	Age (M ± SD or Range)	Female (%)	Sample Type (*n*)	Study Type	Assessment of Pride	Assessment of EDs and/or ED-Related Symptoms	Study Design	Outcome
*Alcaraz-Ibáñez et al., 2019 [44]*	21.25 ± 2.94	41	General population (646)	Quantitative	BSE-FIT	SCOFF; BMI	Cross-sectional	Higher levels of fitness-related pride are associated with higher levels of MEB; the association is stronger in the presence of disordered eating symptoms.
*Chinello et al., 2019 [45]*	40–54	100	AN (5)	Qualitative	Interview	DSM-5, Interview	Cross-sectional	During pregnancy, women in the study experienced a remission of ED-related symptoms; in the post-partum, symptoms re-emerged, and pride feelings connected to pregnancy played a significant role in the remission from AN.
*Cornelius and Blanton, 2016 [46]*	25.80 ± 8.65 20.59 ± 3.40	100 100	Pro-anorexia community (129) ED-recovery websites (133)	Quantitative	Pro-Anorexia Pride Measure	Adapted from standard ED checklists	Cross-sectional	Pro-anorexia endorsement and pride are positively correlated to disordered eating symptoms and negatively correlated to self-esteem.
*Dignon et al., 2006 [47]*	N.s.	87	AN (15)	Qualitative	Semi-structured interviews	DSM-IV	Cross-sectional	Control is one of the trigger factors of the “becoming anorexic” process, while pride (described as a “buzz”) is one of the maintaining factors of the disorder.
*Faija et al., 2017 [48]*	26.99 ± 10.00	100	General population (390)	Quantitative	PEP-S	EDE-Q	Cross-sectional	Global score and all subscales of PEP-S correlated with all subscales of EDE-Q, except for “pride in healthy eating”. PEP-S negatively correlated with self-esteem and authentic pride, and was able to discriminate between participants with high and low ED-related symptoms.
*Faija et al., 2017 [49]*	29.67 ± 12.19	100	AN (21)	Qualitative (GT)	Interview	EDE-Q	Cross-sectional	The onset of anorexia could be linked to the inability to experience pride in food-unrelated contexts; there are different forms of pride in anorexia, each linked to a specific stage of the disorder.
*French et al., 1997 [50]*	Adolescents (no mean age provided)	100	General population (17,159)	Quantitative	Structured interview	Structured interview	Cross-sectional	Low body pride is associated with high dieting frequency, binge-eating behaviors, and purging.
*Howard et al., 2023 [51]*	20–41	100	AN (11)	Qualitative (GT)	Interview	Diagnosis; questions adapted from EDE-Q	Cross-sectional	Past life experiences led to general shame, and perfectionism to alleviate it; self-imposed standards generated more shame. AN becomes a source of pride and a means to numb feelings of shame.
*Lawrence et al., 2024 [52]*	15.57 ± 1.27	NA	LGBTQ (11,083)	Quantitative	Ad hoc self-report questionnaire	Ad hoc self-report questionnaire	Cross-sectional	Low LGBTQ pride is related to higher weight-control and disordered eating behaviors, especially in the presence of bias-based bullying. LGBTQ community is at high risk for developing EDs.
*Ma and Kelly, 2019 [53]*	21.45 ± 2.99	100	AN (23)	Quantitative	SSGS	Diagnosis	Longitudinal (EMA)	Levels of pride increase immediately after exercising, but show a decreasing trend after exercising.
*Marques et al., 2025 [54]*	40.32 ± 14.01	77.3	General population (497)	Quantitative	BASES	EDE-Q; TFEQ	Cross-sectional	Authentic pride is associated with cognitive restraint and possibly identification, while hubristic pride is associated with appearance-related social comparison.
*Mendia et al., 2021 [55]*	25.84 ± 5.36	100	General population (196)	Quantitative	BASES	EAT-26	Cross-sectional	Higher scores in the EAT-26 correlate with higher body-related shame and guilt and lower body-pride (both hubristic and authentic); pride also negatively correlates with anxiety and depression.
*Mendia et al., 2024 [56]*	34.15 ± 9.15 34.84 ± 9.39	0 0	General population (197) General population (163)	Quantitative	BASES	DMS; EDI-3	Cross-sectional	Body pride has no direct effect on EDs in men, but correlates with emotion regulation, which in turn have an effect on ED-related symptoms. Body pride in men does not seem to change between men at risk or not at risk for EDs.
*Mortimer, 2019 [57]*	22.4 ± 1.85	N.s.	Mixed EDs (5) Self-reported as AN, BN, BED	Qualitative	Interview	Diagnosis	Cross-sectional	Anorexia is seen as the most desirable among the EDs; diagnostic crossover is characterized by feelings of shame and pride.
*Muratore et al., 2022 [58]*	34.19 ± 12.02	NA	Transgender/Gender Expansive—TGE (93)	Quantitative	GMSRM (Pride subscale)	EAT-26; DMS	Cross-sectional	Pride is correlated with disordered eating; this could be due to the association between pride and minority stressors (internalized transphobia and non-affirmation).
*Nesbitt et al., 2023 [59]*	35.43 ± 10.09	42.3	MTurk Workers (520)	Quantitative	AHPS; BRPS	EDE-Q	Cross-sectional	Global authentic pride is associated with many indices of mental health (lower anxiety, depression, etc.), while body-related authentic pride is more specifically connected to eating pathology.
*Robinson et al., 2015 [60]*	50	N.s.	AN (8)	Qualitative	Interview	Diagnosis	Cross-sectional	Endurance, as the capacity to live for so long (over 20 years) with AN, is a source of pride for some patients; recovery from the illness could come with an intolerable sense of void.
*Sandoval-Araujo et al., 2024 [61]*	23.5 ± 10.4	72.8	General population (1911)	Quantitative	SSGS	EDE-Q	Cross-sectional	Pride significantly moderates the association between social appearance anxiety (SAA) and ED-related symptoms, i.e., when pride levels were low, the association between SAA and ED-related symptoms was stronger.
*Skårderud, 2007 [62]*	16–39	100	AN (13)	Qualitative	Semi-structured interview	DSM-IV	Cross-sectional	Pride in EDs comes from different sources: 1. Self-control; 2. Being extraordinary; 3. Appearance; 4. Rebellion and protest.
*Thøgersen-Ntoumani et al., 2018 [63]*	21.26 ± 2.76	100	General population (126)	Quantitative	BASES	Compensatory dietary and exercise thoughts and behaviors (adapted from EDE-Q)	Longitudinal (EMA)	Upward appearance comparison is linked with both ED-related symptoms and lower levels of pride (both authentic and hubristic); both types of pride are associated with lower ED-related symptoms.
*Troop, 2016 [64]*	22.2 ± 5.1	100	General population (43)	Quantitative	BPS	CRS; FFQ; food diary	Cross-sectional	Anticipated body pride in the perspective of weight gain or loss can predict levels of dietary restraint and caloric intake; body pride is also linked to several ED-related symptoms.
*von Ranson et al., 2010 [65]*	54.30 ± 9.40	N.s.	Patients with chronic illnesses (60, 21)	Quantitative	SSGS	ESES; EDE-Q	Longitudinal non-randomized clinical trial	Brief CBT psychoeducational intervention can improve ED-related symptoms and feelings of pride in patients with chronic illnesses.
*Watkins and Serpell, 2016 [66]*	25.52	100	General population (52)	Quantitative	Visual Analog Scales	EDE-Q	Longitudinal (EMA)	Fasting considerably increases pride and a sense of achievement in healthy women.

AN = Anorexia Nervosa; BN = Bulimia Nervosa; BED = Binge-Eating Disorder. AHPS = Authentic and Hubristic Pride Scale; BASES = Body and Appearance Self-conscious Emotions Scale; BMI = Body Mass Index; GT = Grounded Theory; BPS = Body Pride and Shame; BRPS = Body-Related Pride Scale; BSE-FIT = Body-related Self-conscious Emotions Fitness instrument; CRS = Cognitive Restraint Scale; DMS = Drive for Muscularity Scale; EAT-26 = Eating Attitude Test—26 Items version; EDE-Q = Eating Disorders Examination Questionnaire; EDI-3 = Eating Disorders Inventory, 3rd edition; EMA = Ecological Momentary Assessment; ESES = Eating Self-Efficacy Scale; FFQ = Food Frequency Questionnaire; GMSRM = Gender Minority Stress and Resilience Measure; MEB = Morbid Exercise Behavior; PEP-S = Pride in Eating Pathology Scale; SSGS = State Shame and Guilt Scale. N.s. = Not specified.

**Table 3:** Summary table for quantitative studies.

	Type of Analysis	Outcome Variable	Effect Size	Direction	Statistical Significance	Sample Size
*Alcaraz-Ibáñez et al., 2019 [44]*	Moderation analysis (Pride × Disordered Eating)	Exercise Addiction	Hubristic β = 0.13	+	*p* < 0.001	646
			Authentic β = 0.16	−	*p* < 0.001	
*Cornelius and Blanton, 2016 [46]*	Moderation Analysis (Pride × Disordered Eating)	Self-esteem	Sample 1 β = 0.20	+	*p* < 0.01	129
			Sample 2 β = 0.14	+	*p* = 0.02	133
*Faija et al., 2017 [48]*	Correlation	EDE-Q	r = 0.62	+	*p* < 0.001	390
*French et al., 1997 [50]*	Regression/Odds Ratio	Dieting	β = 0.17	−	*p* < 0.001	17,159
		Purge yes/no	OR = 0.81	−	95% CI	
*Lawrence et al., 2024 [52]*	/	/	/	/	/	11,083
*Ma and Kelly, 2019 [53]*	Multilevel models	Trends of pride in relation to exercising	Before: r = 0.24	+	*p* < 0.001	23
			After: r = 0.19	−	*p* = 0.001	
*Marques et al., 2025 [54]*	Network analysis	/	/	/	/	497
*Mendia et al., 2021 [55]*	Correlation	EAT-26	Authentic r = 0.20	−	*p* < 0.01	196
			Hubristic r = 0.23	−	*p* < 0.01	
*Mendia et al., 2024 [56]*	Regression	Disordered Eating	Authentic β = 0.09	−	N.S.	197
			Hubristic β = 0.24	−	*p* < 0.001	
			Authentic β = 0.16	−	*p* = 0.04	163
			Hubristic β = 0.25	−	*p* < 0.001	
*Muratore et al., 2022 [58]*	Correlation	EAT-26	r = 0.27	+	*p* < 0.05	93
*Nesbitt et al., 2023 [59]*	Correlation	EDE-Q	Global r = 0.27	−	*p* < 0.01	520
			Body r = 0.36	−	*p* < 0.01	
*Sandoval-Araujo et al., 2024 [61]*	Correlation	EDE-Q	r = 0.47	−	*p* < 0.01	1911
*Thøgersen-Ntoumani et al., 2018 [63]*	Correlation	Restriction and exercise	−0.14 < r < 0.11	/	*p* < 0.01	126
*Troop, 2016 [64]*	Regression	Restraint	β = 0.35	−	*p* < 0.05	43
*von Ranson et al., 2010 [65]*	Change after intervention	/	Cohen’s d = 0.53	/	*p* < 0.01	60
			Cohen’s d = 0.70		*p* = 0.02	21
*Watkins and Serpell, 2016 [66]*	ANOVA for repeated measures	Effects of fasting on pride feelings	Main F = 46.92	+	*p* < 0.05	52
			Interact. F = 12.09	+	*p* < 0.05	

+ = Positive direction of the relation; − = Negative direction of the relation; / = No identifiable direction of the relation.

## Data Availability

No original data were generated or analyzed.

## Supplementary Materials

The following supporting information can be downloaded at: https://www.mdpi.com/article/10.3390/jcm15020522/s1, Table S1: Prisma 2020 Checklist for Systematic Reviews; Table S2: Prima Extension for Scoping Reviews (ScR).
